# Organizing Pneumonia by Paragonimiasis and Coexistent Aspergilloma Manifested as a Pulmonary Irregular Nodule

**DOI:** 10.1155/2011/692405

**Published:** 2011-07-02

**Authors:** In Jae Lee, Jinwon Seo, Dong Gyu Kim

**Affiliations:** ^1^Department of Radiology, Hallym University College of Medicine, Seoul 150-030, Republic of Korea; ^2^Department of Radiology, Hallym University Sacred Heart Hospital, 896, Pyungchon-dong, Dongan-gu, Anyang-city, Gyeonggi-do 431-070, Republic of Korea; ^3^Department of Pathology, Hallym University College of Medicine, Seoul 150-030, Republic of Korea; ^4^Department of Internal Medicine, Hallym University College of Medicine, Seoul 150-030, Republic of Korea

## Abstract

Organizing pneumonia by paragonimiasis and coexistent aspergilloma as a pulmonary nodule is a rare case of lung disease. Its radiographic or CT feature has not been described before in the radiologic literature. We present organizing pneumonia by paragonimiasis and coexistent aspergilloma manifested as a pulmonary irregular nodule on CT.

## 1. Introduction

The CT features of pleuropulmonary paragonimiasis include pleural effusion, hydropneumothorax, pulmonary nodule or air-space consolidation, and cysts [1]. The classical CT feature of aspergilloma is a discrete lesion within the cavities of healed pulmonary tuberculosis and other fibrotic lung diseases [2]. To our knowledge, there was rare radiologic report about organizing pneumonia by pulmonary paragonimiasis and coexistent aspergilloma. We demonstrate organizing pneumonia by paragonimiasis and coexistent aspergilloma manifested as a pulmonary irregular nodule on CT.

## 2. Case Report

**Figure 1:**
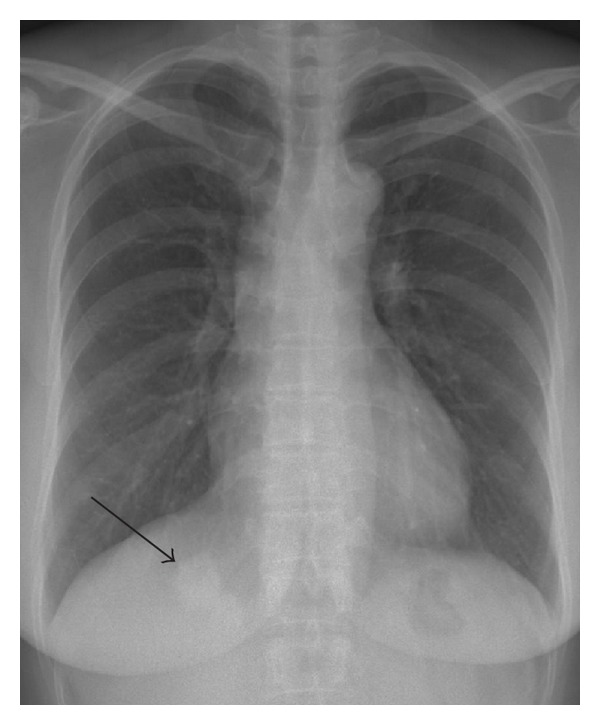
Chest radiograph shows dumbbell-shaped nodule (arrow) in right lung base.

**Figure 2:**
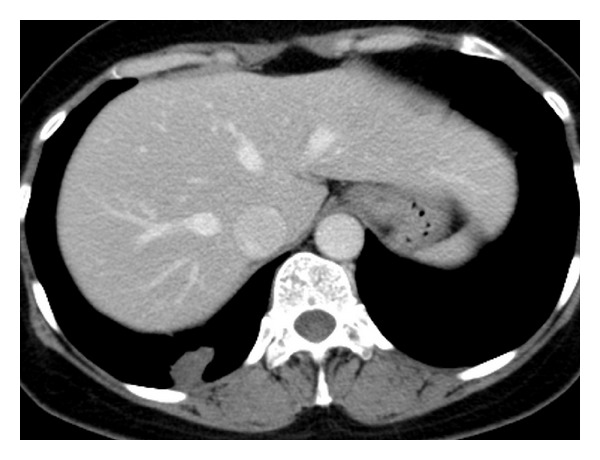
Axial contrast-enhanced CT scan shows irregular 2.5 cm solid irregular nodule with slightly heterogeneously low attenuation without significant enhancement. Note partly broad pleural attachment with adjacent pleural thickening and extrapleural fat thickening.

**Figure 3:**
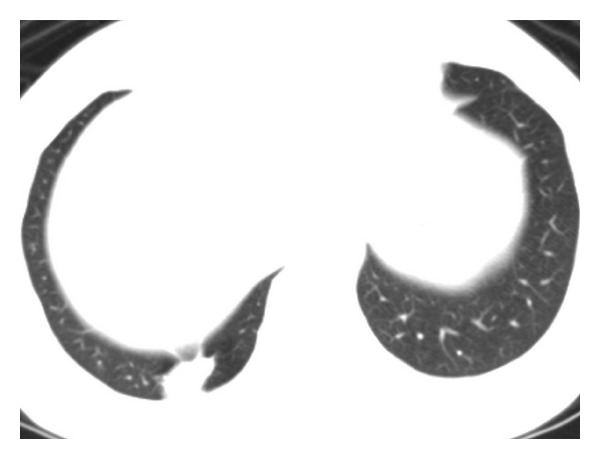
CT scan at lung window setting shows subpleural nodule with pleural tags and partly spiculate margin. Note panlobular emphysema in adjacent area.

A 48-year-old woman was found to have an incidental right lung nodule on a routine chest radiograph (Figure 1). The lesion was dumbbell-shaped nodule in the right lung base. She was asymptomatic including without any chest discomfort. The patient had no history of tobacco smoking, pneumothorax or pleural effusion. Physical examination and blood tests including serum eosinophil count were normal. CT scan of the thorax confirmed the presence of a right lower lobe posterior basal segment nodule (Figures 2 and 3). The lesion was irregular 2.5 cm solid subpleural nodule with focal peripheral ground-glass opacity and partly spiculate margin. The lesion had partly broad pleural attachment and several pleural tags with adjacent pleural thickening and extrapleural fat thickening. The lesion was slightly heterogeneously low attenuation without calcification. There was no significant enhancement of the lesion following intravenous contrast administration. Surrounding panlobular emphysema was also noted. There was no large hilar or mediastinal lymph node, pleural effusion, or pneumothorax. Other lung lesion was not noted on CT. Other respiratory examinations including pulmonary function test and bronchoscopy showed no remarkable finding.

**Figure 4:**
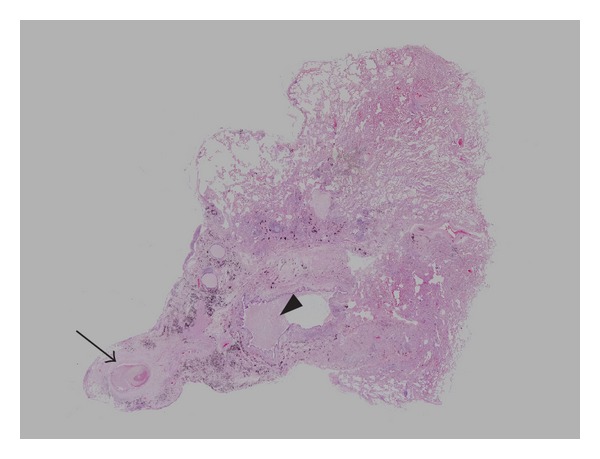
Photomicrograph of H&E stained pathologic specimen from wedge resection of lung shows histologic changes compatible with organizing pneumonia. Note a small subpleural cavitary lesion (arrow) and a dilated small bronchus filled with necrotic materials (arrowhead).

**Figure 5:**
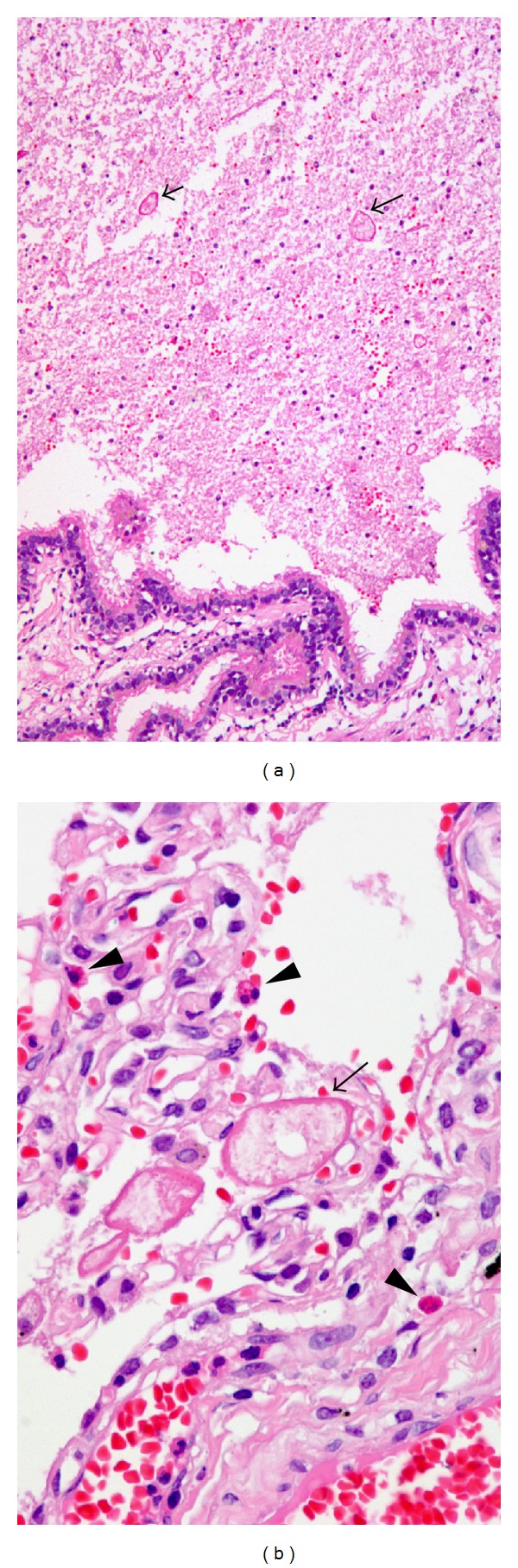
(a) The dilated bronchus is filled with necrotic materials and scattered parasitic eggs (arrows) (H&E, ×100). (b) Scattered degenerated parasitic eggs (arrow) morphologically consistent with ova of *Paragonimus westermani*, intra-alveolar macrophages, and some infiltrating eosinophils (arrowheads) are noted in organizing pneumonia area (H&E, ×200).

**Figure 6:**
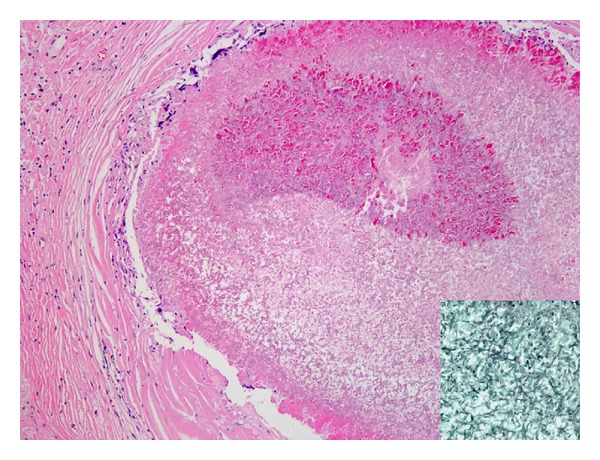
The subpleural cavitary lesion is filled with fungus ball, and the surrounding stroma shows fibrosis and foreign body reaction (H&E, ×100). These fungal hyphae are highlighted on Gomori methenamine silver stain (lower right, ×200) and are morphologically compatible with aspergillus species.

 A wedge resection was performed of the nodule in the posterior basal segment of the right lower lobe. About 2.5 cm soft nodule was detected in this area with pleural adhesion. Yellow punctate spots were also noted in the adjacent visceral, parietal pleura, and diaphragm. On histopathology, peripheral organizing pneumonia by paragonimiasis and coexistent small subpleural aspergilloma was noted (Figure 4). There were dilated peripheral bronchus and terminal bronchiole within the organizing pneumonia. The dilated peripheral bronchus and terminal bronchiole were filled with necrotic material and scattered degenerated parasitic eggs (Figure 5). On the high power field view of microscopic examination, intra-alveolar macrophages, scattered parasitic eggs, and some infiltrating eosinophils were noted. These scattered degenerated parasitic eggs were morphologically consistent with ova of *Paragonimus westermani*. However, there was no living larva in the area of organizing pneumonia. In the peripheral portion of the organizing pneumonia, small subpleural intracavitary aspergilloma was also combined (Figure 6). The aspergilloma was about 2 mm in diameter. Fibrosis and foreign body reaction were also noted in the surrounding pulmonary parenchyma. Final diagnosis was organizing pneumonia by paragonimiasis and coexistent aspergilloma.

## 3. Discussion

Paragonimiasis is an infection caused by the lung fluke *Paragonimus westermani*. The disease is endemic in Southeast Asia and the Far East [1, 3–5]. The common CT findings of pleuropulmonary paragonimiasis include pleural effusion, hydropneumothorax, pulmonary nodules or air-space consolidation, and cysts [1]. Of the nodular lesions, subpleural or subfissural nodule with low attenuation is common [1]. Bronchial wall thickening is also common CT finding, and it reflects inflammatory process along the airway [3, 4]. In this case, the lesion was irregular subpleural solid nodule with internal low attenuation and focal peripheral ground-glass opacity. Considering other report about pulmonary paragonimiasis, this focal peripheral opacity may represent hemorrhage or inflammation in the lesion [4]. On histopathology of this case, multiple degenerated parasitic eggs with chronic inflammation and focal hemorrhage were noted, and this finding may represent low attenuation of the lesion and surrounding focal ground-glass opacity. The degenerated parasitic eggs were morphologically consistent with ova of *Paragonimus westermani*. Organizing pneumonia was developed by paragonimiasis in this case. However, there was rare radiologic report about organizing pneumonia by paragonimiasis. No significant enhancement of the lesion on CT was possibly due to necrotic material within the dilated bronchus and very mild chronic inflammation by degenerated parasitic eggs. Living larva was not detected on histopathologic examination, and it supports no presence of subpleural streaky opacity suggesting worm migration track on CT in this case [1, 3]. It also supports no elevation of serum eosinophil count in this patient. Degenerated parasitic eggs were scattered within dilated bronchi, bronchioles, and alveolar spaces, and bronchial walls were thickened. These findings support inflammatory process along the airway [3, 4]. Pleural or fissural thickening is common in paragonimiasis [1]. In this case, adjacent pleural and extrapleural fat thickening was also noted without pleural effusion. Considering degenerated parasitic worm on histopathology, these findings suggested chronic organizing pneumonia by paragonimiasis. And there was small combined subpleural intracavitary aspergilloma in the peripheral portion of this organizing pneumonia. However, the cavity was not noted on CT. In this case, the cavity could be formed by paragonimiasis considering other radiologic reports about it [1, 3].

Radiologically aspergilloma is a discrete lesion that *Aspergillus fumigatus* colonizes within the cavities of healed pulmonary tuberculosis and other fibrotic lung diseases [2]. The common sites of aspergillomas are upper lobe and lower lobe superior segment [6]. A typical radiologic finding of aspergilloma is a solid, round, or oval mass with soft-tissue opacity within a lung cavity, manifesting an “air crescent sign” without significant enhancement [6]. In this case of intracavitary aspergilloma, air halo was interrupted on radiographs and CT probably due to the limit of radiographic or CT resolution. Moreover the lesion showed no significant enhancement, and it was located in the lower lobe posterior basal segment. The intracavitary aspergilloma was small subpleural lesion in the periphery of organizing pneumonia in this case. Considering the location of the lesion and characteristics of paragonimiasis, the cavity containing aspergilloma could be formed by this parasite. However, there was no radiologic report about aspergilloma following organizing pneumonia by paragonimiasis. Considering the cause of lung lesion and location of the aspergilloma in this case, it is different from the usual features of pulmonary aspergilloma.

In conclusion, organizing pneumonia might be occurred by paragonimiasis and aspergilloma can be combined, and it can be manifested as a pulmonary irregular nodule.
